# Provision of employment-related settlement services and relationship with paid employment for immigrants in Canada

**DOI:** 10.1371/journal.pone.0321927

**Published:** 2025-04-29

**Authors:** Sonja Senthanar, Mieke Koehoorn, Christopher B. McLeod

**Affiliations:** 1 School of Population and Public Health, The University of British Columbia, Vancouver, British Columbia, Canada; 2 Partnership for Work, Health and Safety, The University of British Columbia, Vancouver, British Columbia, Canada; 3 School of Health Sciences, University of Northern British Columbia, Prince George, British Columbia, Canada; University of Saskatchewan, CANADA

## Abstract

Immigrant workers are overly represented in high risk and precarious jobs that are not commensurate with their background, skills and experience. Some evidence exists to suggest that access to employment-related (ER) supports and services in the community can help leverage job opportunities. This study examined the association between use of government-funded ER services and paid employment of immigrants to Canada. ER service records were linked with immigration and taxation records for individuals who immigrated to Canada between 2015–2017. The cohort was restricted to immigrants with no paid employment in their year of landing to examine the direct impact of ER service (measured as any ER service, intensity of service and type of ER service) on subsequent employment. The outcome of subsequent employment in the year following ER service provision was estimated using adjusted logistic regression models. Immigrants displayed a higher odds of paid employment the year following the ER service for individuals that accessed any ER service (OR = 1.57; 95% CI, 1.49 to 1.65), across the measures of ER intensity (ranging from OR = 1.26; 95%CI, 1.09 to 1.45 at the lowest intensity percentile to OR = 2.21; 95% CI, 1.95 to 2.52 at the highest intensity percentile), and by type of ER service (essential skills and aptitude training, OR = 1.35; 95% CI, 0.93 to 1.96; short-term intervention, OR = 1.57; 95% CI, 1.49 to 1.66; long-term intervention, OR = 1.45; 95% CI, 1.07 to 1.95) compared to immigrants who did not access ER services. This study finds that access to an ER service is associated with paid employment. It highlights key services that should be promoted to facilitate employment integration but also potential barriers to integration that warrant further investigations.

## Introduction

Immigration plays a critical role in the Canadian economy, namely, to address labour market shortages in numerous sectors. It is estimated that roughly one-quarter of Canadian workers are immigrants and immigration will continue to contribute significantly to population growth over the next decade [1]. Immigrants arrive to Canada through various immigration classification programs, the major ones being as economic immigrants, family members, and refugee classifications. Briefly, economic immigrants are selected via a points system that assigns a score (range 0–1200) based on education, work experience, and language proficiency in either English or French. Applicants with higher scores are more likely to be invited to apply for immigration through the economic classification stream. Applicants with family in Canada who can provide financial support (up to 10 years) can resettle through specific family reunification programs, and applicants seeking refuge and protection due to ongoing violence and civil unrest in their host countries can resettle through a series of refugee programs (these programs are funded by the federal government, privately sponsored or through a joint initiative between the government and private sponsoring groups).

On arrival to Canada, employment integration is at the forefront of resettlement for an immigrant worker in terms of financial security and making contributions as a member of society in a new country. For the purposes of the current study, we define the successful employment integration of immigrants as the unimpeded entry and participation of immigrants into the labour market in their new country and in employment in occupations that are like those previously held [2]. However, employment integration is a complex process that is influenced by several intersecting factors. Prior research on employment integration has found that new immigrants have an employment rate disadvantage compared to Canadian-born workers that persists over time despite key factors such as level of education that might be expected to facilitate this process [3,4]. In 2016, for example, the proportion of immigrants in high-skill jobs requiring a bachelor’s degree was roughly 20 percentage points less than their Canadian-born counterparts [5]. When examined by immigrant classification (primary reason for immigration to Canada), economic immigrants tend to have higher employment rates and earnings growth than other immigrant groups (e.g., earning in the first full year rose 39% among economic immigrants between 2010 and 2018 compared to 27% for family sponsored and 9% for refugees) [5].

A substantive body of literature has documented the persistent challenges that immigrants encounter in finding employment in Canada [6–10]. These challenges range from individual-level barriers including a lack of English or French language proficiency and Canadian work experience that is preferred by employers [6,11], social barriers such as limited affordable childcare and attitudes with regards to gender and work [12,13], and structural features of the labour market that create barriers such as credentialing bodies and organizational hiring practices that may not recognize immigrant workers’ education, training and prior experiences [6,14]. These challenges intersect to push newcomers into low-wage and insecure ‘survival’ jobs [9,15]. For example, in Premji and Shakya’s (2017) study on underemployment of immigrant women in Toronto, Canada, women describe a context of being a primary caregiver to their children coupled with limited transportation options (to attend language classes) and racialized discrimination that viewed them as less valuable in the labour market [16]. These challenges are worsened by inaccessibility to resources (e.g., interpreters or translators) and a lack of social connections as a newcomer [6,17]. As a result, for many immigrants, securing any job upon arrival may be a favourable outcome to that of unemployment and may lead to occupational mobility.

To mediate labour market integration challenges, immigrants often participate in employment-related (ER) services (1 of 6 major categories of settlement services provided to newcomers). These services are funded by the federal government, Immigration, Refugees and Citizenship Canada (IRCC), and delivered by a network of community-based, non-profit settlement organizations. There are nearly 3600 settlement organizations across Canada and roughly 46% provide employment-related services [18]. Current settlement services in Canada are organized and funded on a needs-basis; that is, services are designed and delivered to align with the changing needs of immigrants [19].

ER services are a trusted resource for newcomers to help gain the knowledge and skills to enter the Canadian labour market [20]*.* These services include a range of activities such as help with resume writing, networking and mentorship opportunities with employers, job bridging programs, and skills and aptitude training (e.g., computer use, interpersonal skills). Between 2016 and 2020 in Canada, ER services were the third most accessed government service by adult immigrants. They were most used by refugees (25%) and least used by family sponsored immigrants (6%) [21]. Studies examining employment outcomes following use of ER services have been mixed [13,22–24]. For example, a survey focusing on skilled economic immigrants found that participation in long-term interventions (e.g., fixed-term work placement with an employer) had a significant positive effect on labour market participation as measured by on-going full-time employment [25]. Qualitative investigations have found that immigrant workers appreciate employment-related settlement services but that the resulting employment opportunities are less desirable involving deskilled and low-wage work [13,24]. Collectively, the provision and use of ER services has grown in Canada but in-depth examinations of its effectiveness in securing paid employment for new immigrants is limited and few studies have employed a multivariable analysis to isolate the effect of ER services on employment by adjusting for potential confounders of this relationship.

This study examined the effectiveness of government-funded ER service defined as provision of any ER service, intensity of ER service, and type of ER service in securing paid employment (yes/no) for new immigrants who were not employed in their year of landing in Canada. The available ER data for this study precluded any measure of the quality of the paid employment (e.g., skill level) associated with ER services and consequently is not examined in this analysis. A focus on immigrants who were not employed in their landing year was intentionally selected to examine the direct effect of ER service use on paid employment in subsequent years for the immigrant population most in need of these types of settlement services and for which services were hypothesized to have an important temporal effect for longer term employment trajectories.

## Methods

### Data sources

De-identified administrative data from The Longitudinal Immigration Database (IMDB) and The Settlement Services Database were linked at the individual level to construct a cohort of immigrants who accessed employment-related settlement services and with information on employment wages and salaries. Population-level data from The IMDB and Settlement Services Database are collected by the federal government and managed by Statistics Canada on behalf of a federal-provincial consortium led by IRCC.

The IMDB is a database that combines administrative files on immigrants admitted to Canada since 1980 with their annual tax files. It is a comprehensive source of data on the economic behavior of the immigrant tax filer population in Canada and allows for the analysis of labour market trends over time [26]. The Settlement Service Database derives information from the Immigration Contribution Agreement Reporting Environment (iCARE) that collects and tracks settlement services accessed by immigrants since 2013. The Settlement Service Database is comprised of 15 files (for different types of services in Canada) of which the current study used the one relating to employment-related service use. Linkage and access to the novel IMDB-Settlement data was made available by Statistics Canada to a select number of investigators for approved research questions.

### Study sample

The study sample for the current analyses included all immigrants, aged 18–75 years, who landed in Canada and accessed an ER service over a three-year period, between 2015 and 2017, and who did not have paid employment in their year of landing. Immigrants aged 18–75 years old were included to reflect the characteristics of the Canadian labour force during the study period and immigration labour force data that suggests both younger and older workers are actively seeking employment. As well, the IMDB-Settlement dataset captures immigrants who had engaged with employment-related services at some point since arriving to Canada, providing a strong indicator that the included sample of 18–75-year-olds are actively seeking work.

### Study variables

The primary outcome variable was paid employment as recorded annually in the IMDB tax files as salaries from employment. Paid employment was defined as a dichotomous outcome of no paid employment (wages and salaries <$1,000 CA) in the year following ER service use, or yes for paid employment (wages and salaries>=$1,000 CA) in the year following ER service use. A $1000 CA cut-point was used as recommended when using tax data and to reduce the possible inconsistency in the tax-filing patterns of low earners [27].

Explanatory variables were derived from the ER services file that included 37 variables of interest. Upon exploration of the variables, the effect of ER service was analyzed in three ways: 1) the use of any ER service, defined as ‘yes’ or ‘no’ in the year of landing; 2) intensity of ER service use, defined as the sum of hours spent across ER services in the year of landing; and 3) type of ER service grouped into four pre-assigned categories by Statistics Canada as essential skills and aptitude training, short-term intervention, long-term intervention, and other supports received in year of landing. Briefly, 1) Essential skills and aptitude training included computer skills, document use, interpersonal and workplace culture, leadership, and numeracy skills training; 2) Short-term intervention included employment counselling, resume/CV matching and referral services, and networking opportunities; 3) Long-term intervention included work placement opportunities, mentoring services, and preparation for licensure/certifications; and 4) Other supports included child-minding assistance, crisis counselling, provisions for disabilities to attend ER services training, and interpretation, translation and transportation services.

Potential covariates of the association between ER service use and subsequent employment included immigration classification, defined as an individual who immigrated to Canada as an economic, family member or refugee/other immigrant; age at time of arrival categorized into 10-year age groupings between 18–75 years; gender coded as male or female; province of residence, excluding Quebec (information on settlement services is not collected) and combining the four Atlantic provinces and the three Territories due to small sample sizes; official language proficiency categorized as English only, French only, English and French, or neither English nor French; family status defined as principal applicant or spousal/dependent applicant; and marital status defined as single, married/common-law, or separated/divorced/widowed.

### Data analysis

The distribution of the study variables was examined for immigrants with and without paid employment in the year following landing. Crude associations between each categorization of ER service and paid employment were measured using bivariable logistic regression models. Confounding was investigated by adding each additional variable of interest to the bivariable logistic models one by one. A series of multivariable logistic regression models were then used to examine the ER service-paid employment, ER intensity-paid employment, ER type-paid employment relationships, adjusted for confounders. The multivariable logistic regression analyses are presented as odds ratios (ORs) with 95% confidence intervals (CI) where an OR greater than ‘1’ specifies an increased odds of paid employment in the year following ER service.

Access to and use of data was conducted in the Research Data Centre at the University of British Columbia. Ethics approval was covered by the University of British Columbia Policy No. 89 and the Tri-Council Policy Statement: Ethical Conduct for Research Involving Humans (article 2.2) governing the use of publicly available survey data [28]. All statistical analyses were conducted using SAS 9.4 [29].

## Results

### Descriptive results

The characteristics of the study sample are summarized in Table 1. Overall, there was an equal distribution of immigrants by paid employment status the year after landing. Compared with immigrants who were not employed, immigrants who were employed were more likely to have accessed an ER service in their year of landing (36.6% versus 48.3%), have arrived as an economic immigrant (41.0% versus 50.1%), were men (39.0% versus 49.9%), have a higher level of education (33.3% versus 37.8% with a Bachelor’s degree*,* and be younger (48.3% versus 58.7% between the ages of 18–34 years).

**Table 1:** Socio-demographic and socio-economic characteristics of immigrants arriving to Canada between 2015 and 2017 by paid employment status the year after landing.

	No paid employment in year after landing (n = 13,925)	Paid employment in year after landing (n = 13,110)
Provision of ER service in landing year		
No	8825 (63.4%)	6775 (51.7%)
Yes	5095 (36.6%)	6330 (48.3%)
Immigration classification upon landing in Canada		
Economic	5745 (41.3%)	6560 (50.1%)
Family member	2590 (18.6%)	2780 (21.2%)
Refugee/other	5585 (40.1%)	3765 (28.7%)
Gender		
Men	5425 (39.0%)	6535 (49.9%)
Women	8495 (61.0%)	6565 (50.1%)
Age at time of landing in years		
18–24	1390 (10.0%)	1570 (12.0%)
25–34	5330 (38.3%)	6120 (46.7%)
35–49	5840 (41.9%)	4785 (36.5%)
50–64	1150 (8.3%)	595 (4.5)
65 +	215 (1.5%)	35 (0.3%)
Family status at time of landing		
Principal applicant	8850 (63.6%)	9470 (72.3%)
Spousal/dependent applicant	5070 (36.4%)	3635 (27.7%)
Marital status at time of landing		
Single	2110 (15.2%)	3250 (24.8%)
Married/common-law	11395 (81.8%)	9580 (73.1%)
Separated/divorced/widowed	420 (3.0%)	270 (2.1%)
Destination province or region at time of landing		
Atlantic/Territories	1245 (8.9%)	950 (7.2%)
Ontario	6845 (49.2%)	6135 (46.8%)
Manitoba	900 (6.5%)	1165 (8.9%)
Saskatchewan	420 (3.0%)	415 (3.2%)
Alberta	3145 (22.6%)	3095 (23.6%)
British Columbia	1365 (9.8%)	1350 (10.3%)
Level of education at time of landing		
None	730 (5.2%)	420 (3.2%)
Secondary or less	4255 (30.6%)	3170 (24.2%)
Some postsecondary	1065 (7.7%)	1055 (8.1%)
Bachelor’s degree	4630 (33.3%)	4960 (37.8%)
Masters or some post-graduate	2935 (21.1%)	3335 (25.4%)
Doctorate	305 (2.2%)	165 (1.3%)
Official language proficiency		
English only	8915 (64.0%)	9985 (76.2%)
French only	325 (2.3%)	360 (2.7%)
Both English and French	245 (1.8%)	215 (1.6)
Neither English nor French	4440 (31.9%)	2550 (19.5%)

### Modeling results

In adjusted multivariable logistic regression, the odds of employment in the year following landing was higher for immigrant workers who used any type of ER service compared with immigrant workers who did not use an ER service in their year of landing (OR = 1.57; 95% CI, 1.49 to 1.65) (Table 2). When examined by ER intensity, there was increasing odds of employment as length of time spent across ER services in the year of landing increased. For example, the odds of employment were 1.26 (95% CI, 1.09 to 1.45) at the lowest percentile of intensity of ER service and rose to 2.21 (95% CI, 1.95, 2.52) at the highest percentile of intensity, compared to immigrant workers who did not access ER services. The largest effect for subsequent employment was seen from the 80^th^ percentile onward of the intensity distribution where immigrant workers may be spending upwards of 100 hours in ER services in the given landing year. For type of ER service accessed, the odds of employment in the year following landing were higher for immigrant workers who accessed essential skills and aptitude training (OR = 1.35; 95% CI, 0.93 to 1.96), a short-term intervention (OR = 1.57; 95% CI, 1.49 to 1.66) or a long-term intervention (OR = 1.45; 95% CI, 1.07 to 1.95), compared to those who did not access ER services. Conversely, there was a reduced odds of subsequent employment for immigrant workers who accessed other ER services (OR = 0.87; 95% CI, 0.76 to 1.00).

**Table 2:** Multivariable logistic regression models for the association between ER service-paid employment, ER intensity-paid employment, and ER type-paid employment (no versus yes in the year following ER service) for new immigrants to Canada without employment in their year of landing, 2015–2017.

OR (95% CI)			
	Model 1	Model 2	Model 3
Provision of any ER service			
No	Ref.		
Yes	1.57 (1.49, 1.65)		
Intensity of ER service (hours spent)			
None		Ref.	
10^th^		1.26 (1.09, 1.45)	
20^th^		1.39 (1.26, 1.52)	
30^th^		1.15 (0.89, 1.48)	
40^th^		1.40 (1.26, 1.56)	
50^th^		1.46 (1.20, 1.79)	
60^th^		1.53 (1.36, 1.73)	
70^th^		1.54 (1.36, 1.73)	
80^th^		1.85 (1.63, 2.10)	
90^th^		2.00 (1.75, 2.28)	
100^th^		2.21 (1.95, 2.52)	
Type of ER service			
None			Ref.
Essential skills and aptitude training			1.35 (0.93, 1.96)
Long-term intervention			1.45 (1.07, 1.95)
Short-term intervention			1.57 (1.49, 1.66)
Other support			0.87 (0.76, 1.00)
Immigration classification of worker			
Economic	Ref.	Ref.	Ref.
Family member	1.01 (0.93, 1.09)	1.03 (0.95, 1.11)	1.01 (0.94, 1.10)
Refugee/other	0.63 (0.58, 0.68)	0.64 (0.59, 0.69)	0.63 (0.58, 0.68)
Sex			
Men	Ref.	Ref.	Ref.
Women	0.57 (0.54, 0.60)	0.57 (0.54, 0.60)	0.57 (0.54, 0.60)
Age at time of landing			
65 +	Ref.	Ref.	Ref.
18–24	7.63 (5.32, 10.95)	7.46 (5.20, 10.71)	7.50 (5.23, 10.77)
25–34	7.37 (5.18, 10.48)	7.22 (5.07, 10.26)	7.29 (5.13, 10.37)
35–49	5.55 (3.90, 7.89)	5.42 (3.81, 7.71)	5.53 (3.89, 7.86)
50–64	3.72 (2.59, 5.35)	3.66 (2.55, 5.26)	3.75 (2.61, 5.39)
Family status at time of landing			
Principal applicant	Ref.	Ref.	Ref.
Spousal/dependent applicant	0.76 (0.71, 0.80)	0.76 (0.72, 0.81)	0.76 (0.71, 0.81)
Marital status at time of landing			
Single	Ref.	Ref.	Ref.
Married/common-law	1.40 (1.17, 1.68)	1.40 (1.17, 1.68)	1.41 (1.18, 1.69)
Separated/divorced/widowed	0.83 (0.70, 0.98)	0.83 (0.70, 0.98)	0.83 (0.70, 0.98)
Destination province			
Atlantic/Territories	Ref.	Ref.	Ref.
Ontario	1.08 (0.98, 1.19)	1.10 (1.00, 1.21)	1.10 (0.99, 1.21)
Manitoba	1.65 (1.45, 1.87)	1.65 (1.45, 1.87)	1.66 (1.46, 1.89)
Saskatchewan	1.24 (1.05, 1.46)	1.22 (1.03, 1.44)	1.24 (1.04, 1.46)
Alberta	1.19 (1.08, 1.33)	1.21 (1.09, 1.35)	1.20 (1.08, 1.34)
British Columbia	1.30 (1.15, 1.46)	1.32 (1.17, 1.49)	1.32 (1.17, 1.48)
Level of education			
None	Ref.	Ref.	Ref.
Secondary or less	1.22 (1.06, 1.40)	1.21 (1.06, 1.40)	1.21 (1.06, 1.40)
Some postsecondary	1.40 (1.19, 1.64)	1.40 (1.19, 1.64)	1.40 (1.20, 1.65)
Bachelor’s degree	1.23 (1.07, 1.42)	1.22 (1.06, 1.40)	1.23 (1.06, 1.41)
Masters or some post-graduate	1.16 (1.00, 1.34)	1.13 (0.98, 1.31)	1.15 (0.99, 1.33)
Doctorate	0.61 (0.48, 0.78)	0.61 (0.48, 0.77)	0.61 (0.48, 0.78)
Official language proficiency			
Both English and French	Ref.	Ref.	Ref.
English only	0.99 (0.84, 1.16)	0.98 (0.84, 1.15)	0.99 (0.84, 1.16)
French only	0.74 (0.61, 0.89)	0.74 (0.61, 0.89)	0.73 (0.61, 0.89)
Neither English nor French	0.61 (0.56, 0.65)	0.61 (0.56, 0.65)	0.61 (0.57, 0.65)

Abbreviations: CI, confidence interval; OR, odds ratio; ref, reference category

For the covariates, immigrants arriving as refugee/other classifications, women, spousal and dependent immigrants, individuals that did not have language proficiency in either English or French, and individuals that did not have a partner had a reduced odds of employment the year following landing. Immigrants that were working age (18–64 years) or resided in populous provinces had a higher odds of employment. Surprisingly, immigrants that arrived with a doctorate degree had a reduced odds of employment compared to immigrants with no known educational attainment.

### Discussion

In this study, immigrant workers who were not employed in their landing year but accessed an employment-related service in that year had a one and half (for any service and short-term service type) to two times (highest ER intensity percentile) the odds of paid employment the year following service provision, compared with immigrants who were not employed in their landing year and who did not access an employment-related service in that year. The finding of higher odds of paid employment was not found for immigrant workers who accessed ‘other support’ as a type of ER service. The IMDB-Settlement Database characterizes other support as childminding, crisis counseling, provisions for disabilities, interpretation, translation, and transportation-related supports. By way of explanation for this finding, it is possible that immigrants accessing other supports have additional challenges to employment integration compared to workers who access essential skills and aptitude training or short- and long-term interventions. For example, research examining individual (e.g., lack of language proficiency) and social barriers (e.g., childcare) find that these barriers are often intersecting and prevent immigrant workers from actively and consistently searching for work [13,30,31]. Providers of ER services may find it helpful to know that those accessing other supports provides an indicator or a surrogate indicator of immigrants with additional barriers to employment no matter the other types or intensity of ER services provided.

Federally funded employment-related services are provided to newcomers at no cost to help in labour market participation and gaining economic security in Canada. These services are diverse and range in quality from discrete sessions focused on improving essential workplace skills, short-term sessions focused on resume screening or employment counseling to longer-term interventions focused on one-on-one employer mentorship. With the exception of other supports provided, the current study found that immigrants accessing any of these types of services saw a positive effect on their employment. The findings corroborate prior research conducted in the Canadian provinces of Alberta and Manitoba where half the respondents reported that ER services were helpful with 60% finding FT employment in Alberta and 41% finding FT employment in Manitoba [32]. A commonly cited issue by immigrant workers in this study, however, was suitability of the employment to their prior skills and qualifications. While the current study was not able to assess the quality or nature of employment due to data limitations, some qualitative research in this area has been able to tease apart the ER service type-employment relationship. For example, Senthanar et al. 2019 found that settlement organizations often had pre-existing ties with employers in the community who were seeking deskilled and low-wage labour that was offered to immigrants through subsidized work placements [13]. Similarly, in their study on employment integration of immigrants and refugees, Kosny et al. 2020 found that working conditions were often unsafe and exploitative despite immigrants expressing appreciation for ER services such as employer matching [24]. In both prior studies by Senthanar and Kosny, settlement organizations focused on helping immigrant workers gain Canadian work experience with the intent of eventually leading to better positions.

The intensity of ER service use across the varied services is relative to the time a newcomer is willing and able to dedicate to participation in such services. However, the current study found that immigrants who spent any amount of time in ER services, from the 10^th^ percentile (about 1–2 hours) to 80^th^ percentile and higher (upward of 100+ hours) were effective to securing paid employment, although the effect was greatest with more hours. There are a few potential explanations for this finding. First, immigrant workers are able to strategically chose an ER service or services suited to their needs rather than participate in a hierarchy of services as seen with language classes [33] and thus, maximize their efforts. Second, the ability to choose when and for how long an immigrant wants to participate in an ER service is advantageous especially in the case where individuals have competing demands in and outside the home. For example, immigrant women often experience “double workday” when paid, whether formal or informal, and unpaid work such as high load of household and caregiving work intersect [34,35]. Thus, flexibility in ER service participation provides immigrants with the empowerment to do what makes sense for their schedules which has been shown to improve employment status [36]. Third, the finding of two times the odds of paid employment for immigrants who spent the longest duration in ER services is intuitive as these individuals not only benefitted from different ER services but repeated contacts with service providers to maximize skillsets and opportunities. Employment service providers are often criticized for inadequate service provision due to funding and staffing constraints and high workloads [37–39] that limit a newcomer’s tailored and one-on-one interactions with ER services. The current findings lend support to increased government funding and staffing for more intensive participation, leading to better employment outcomes.

Compared with immigrants who arrive to Canada as economic immigrants, refugees/other classification immigrants experienced lower odds of paid employment which is consistent with an extensive body of evidence around refugee integration [6,40–42]. While the main effects of immigration classification were not described here, the role of immigration classification should be considered in future analyses of the ER service-paid employment relationship to ascertain whether refugees as a subgroup may differentially benefit from ER services. The finding of a reduced odds of paid employment for doctorate degree holders who access an ER service may be explained, in part, by the design of employment programs that are usually geared toward less skilled jobs that are not commensurate with the skill level and training of these workers but also by a potentially higher reservation wage (wage at which an individual is willing to work) [43]. Highly educated immigrants may opt to explore other pathways to education-occupation matched employment (e.g., additional training and upskilling) following ER services that may translate to a break in participation in a paid position.

### Strengths and limitations

Strengths of this study included the use of a large sample of newly arrived immigrants accessing employment-related settlement services linked with annual tax records for access to novel linked data for the investigation of ER service provision and paid employment. The pilot IMDB-Settlement linkage provided detailed data on a number of ER service measures that allowed for examination by any service, type of service and intensity of service to consider effectiveness of these services on paid employment in different ways. The methodological decision to include immigrants with no paid employment in their year of landing was employed to focus on service provision in that year as an important window of opportunity for quick economic integration with potential longer term positive employment pathways. Further the linkage to tax records provided a valid and reliable indicator of employment status.

There are a few limitations to consider. First, the ER database is by definition comprised of a sub-set of immigrants who access ER services at least once since landing in Canada and excludes the sub-set of immigrants who never access ER services. However, to enable the investigation of the main research questions on the effect of ER services on subsequent employment, the study sample in the ER database included those who were not employed in their landing year and then compared those who access at least one service in this landing year with those who did not access a service in this landing year (but would have accessed a service at any time subsequently). While immigrants who access an ER service at any time represent a distinct group it does not negate the effect of ER services on employment observed among this sub-population in the current study. Those who never access ER services are worthy of further investigation with regards to their employment status and as part of a complete picture of employment related supports for new immigrants (e.g., access to resources, awareness and knowledge about settlement services, accessibility to services), but this investigation necessitates different linked databases than those available to the investigators. Second, due to the nature of the dataset, the investigators were not able to investigate the relationship between ER service variables and the type or quality of employment. This study demonstrated the utility of using the linked IMDB-Settlement dataset for employment research and evidence of the effectiveness of employment services on any paid employment provides a necessary and foundational piece of evidence to inform and drive more detailed economic analyses. Future research beyond this initial investigation could utilize the data in the tax records to look at outcomes such as sustained employment over time and level of income or income growth. Third, the current analyses highlighted a strong effect for age that should be examined further with the inclusion of interaction terms with age (and education or language) or stratified models by age groups to tease out the effects of ER service for younger compared to older workers, for example. Finally, a 1-year follow-up for paid employment provides a preliminary view of employment integration. As settlement data continues to be collected and integrated with tax records, extended measures of ER services (e.g., continued participation) and paid employment such as continued employment (year-to-year) and employment trajectories can capture the full extent of the relationship.

## Conclusion

Findings from this study indicate that employment-related settlement services are associated with immigrants’ economic integration. In particular, access to any ER service is beneficial to subsequent paid employment but the type of ER service and intensity of these services matter. The findings highlight the importance of continued provision of these federally funded services, considerations of how to make services more accessible to increase time spent in these services, and longer-term supports for those with additional barriers such as those related to childcare. Integration of information on ER services in information and orientation sessions, which are the most accessed settlement service overall for new immigrants [21], is recommended to promote earlier and continued participation in ER services.
